# The intestinal microbiome associated with lipid metabolism and obesity in humans and animals

**DOI:** 10.1111/jam.15740

**Published:** 2022-08-08

**Authors:** Zonghui Jian, Li Zeng, Taojie Xu, Shuai Sun, Shixiong Yan, Sumei Zhao, Zhengchang Su, Changrong Ge, Yunmei Zhang, Junjing Jia, Tengfei Dou

**Affiliations:** ^1^ Yunnan Provincial Key Laboratory of Animal Nutrition and Feed Yunnan Agricultural University Kunming People's Republic of China; ^2^ The Chenggong Department Kunming Medical University Affiliated Stomatological Hospital Kunming People's Republic of China; ^3^ Yunnan Key Laboratory of Stomatology Kunming People's Republic of China; ^4^ Department of Bioinformatics and Genomics, College of Computing and Informatics The University of North Carolina at Charlotte Charlotte North Carolina USA; ^5^ Department of Cardiovascular, The First People's Hospital of Yunnan Province The Affiliated Hospital of Kunming University of Science and Technology Kunming People's Republic of China

**Keywords:** intestinal microbiota, human and animal, lipid metabolism, short‐chain fatty acid, exercise and diet, metabolic disorder, obesity

## Abstract

Intestinal microbiota is considered to play an integral role in maintaining health of host by modulating several physiological functions including nutrition, metabolism and immunity. Accumulated data from human and animal studies indicate that intestinal microbes can affect lipid metabolism in host through various direct and indirect biological mechanisms. These mechanisms include the production of various signalling molecules by the intestinal microbiome, which exert a strong effect on lipid metabolism, bile secretion in the liver, reverse transport of cholesterol and energy expenditure and insulin sensitivity in peripheral tissues. This review discusses the findings of recent studies suggesting an emerging role of intestinal microbiota and its metabolites in regulating lipid metabolism and the association of intestinal microbiota with obesity. Additionally, we discuss the controversies and challenges in this research area. However, intestinal micro‐organisms are also affected by some external factors, which in turn influence the regulation of microbial lipid metabolism. Therefore, we also discuss the effects of probiotics, prebiotics, diet structure, exercise and other factors on intestinal microbiological changes and lipid metabolism regulation.

## INTRODUCTION

Obesity is induced by the accumulated signals that regulate energy balance. The main factors that affect energy balance are feeding behaviour and energy intake, intestinal energy absorption, energy expenditure and energy storage (Bauer et al., 2016). The association of obesity with type 2 diabetes (T2D) mellitus, cardiovascular disease and decreased longevity makes it a global health problem (Jia et al., 2009). In addition, multiple studies have shown a strong link between COVID‐19 and obesity. Obese patients with COVID‐19 have higher rates of hospitalization, more severe disease progression and worse clinical prognosis than nonobese patients (Ritter et al., 2020). Many factors for obesity have been identified, which include both environmental factors and genetic factors; however, the genetic variations related to obesity could explain only 5% of the variation in body mass index (BMI), and other factors (environment, diet and lifestyle) also explained the genetic contribution to obesity (Ni et al., 2021). Therefore, intestinal microbiota and its metabolites may be the cause of obesity and its metabolic complications and have received extensive attention of scholars. Several pathological conditions including metabolic psycho‐immune diseases and cancers have been associated with changes in the structure and function of intestinal microbiota (Wang et al., 2020). Increasing evidence indicates that the interaction of intestinal microbiota with host genetics, diet and other environmental factors may affect lipid metabolism of the host and lead to obesity (Moran‐Ramos et al., 2017; Tokarek et al., 2021). Changes in the diversity or structure of the gut microbiome, known as dysbiosis, may affect metabolic pathways and lead to metabolic disorders such as obesity, T2D and nonalcoholic fatty liver disease (NAFLD) (Chakaroun et al., 2020; Vallianou et al., 2019). Most studies have shown that the gut microbiota in obese people are less diverse and less abundant than that in lean people, and low gut microbiota diversity and decreased gut microbiota richness are associated with an increased relative risk of obesity (Ferrarese et al., 2018). A key challenge in the field of intestinal microbiology and obesity research is exploring the role of intestinal microbial metabolites such as short‐chain fatty acids (SCFAs, butyric acid, acetic acid, and propionic acid), biliary saline hydrolyase (BSH), trimethylamine‐*N*‐oxide (TMAO) and the microbial component lipopolysaccharide (LPS) in lipid metabolism (Canfora et al., 2019). The gut microbiome has the ability to degrade the indigestible components of our diet, leading to the breakdown of complex carbohydrates such as plant polysaccharide proteins and small amounts of fat. Therefore, diet (high‐fat diet and the diets rich in probiotics, prebiotics and synbiotics) and lifestyle (exercise and intermittent fasting) also affect changes in gut microbiota, which in turn increases the risk of obesity and leads to disordered lipid metabolism in the host.

In this review, we discuss advances in research on the intestinal microbiome diversity in humans and animals and elaborate on the mechanisms of lipid metabolism regulation by the intestinal microbiome and its metabolites. In addition, we discuss the effects of diet, exercise and other factors on intestinal microbe‐mediated regulation of lipid metabolism. Considering the global obesity epidemic that is necessitating the search for better intervention strategies, including harnessing the health benefits of some of the gut microbiome and its metabolites, we also discuss the health‐promoting role of probiotics, prebiotics and synbiotics and their impact on the risk of intestinal microbiome disorders and obesity.

## INTESTINAL MICROBES IN HUMANS AND ANIMALS

The intestinal microbiome can be considered an additional organ of the host that exhibits significant dynamic changes and strong impacts on physiology and pathology. This community consists of at least 10^13^ bacterial species, comprising mainly anaerobic bacteria. Intestinal micro‐organisms are distributed in various segments of the host intestine; however, they mainly colonize the large intestine. Although the small intestine contains only 10^3^–10^7^ microbial cells per gram of contents, the diversity and abundance of microbial flora in the small intestine are much lower than those in the large intestine (Donaldson et al., 2016). By comparing poundage overlap at the gene sequence level, more than 80% of the genes in each species were found to be unique; only a small proportion of genes (~0.5%) was found to be shared by chicken and pig (~0.8%). Chicken and pigs share fewer common microbial genes than chicken and humans (~10%) or pigs and humans (~10%) (Huang et al., 2018).

Colonization of the human intestinal microbiome begins with rapid expansion of bacterial diversity at birth, characterized by an ever‐changing bacterial assemblage, which becomes relatively stable in adulthood (Yatsunenko et al., 2012). This diverse ecosystem is formed by early life events; however, over time, it can evolve through interactions among its components and with exogenous or endogenous factors (Ghazalpour et al., 2016). A recent human gut microbiome study provided a new genome set of the Asian human gut microbiome, an updated Human Reference Gut Microbiome (HRGM). HRGM contains 232,098 nonredundant genomes for 5414 representative prokaryotic species including 780 that are novel genes, >103 million unique proteins and >274 million single‐nucleotide variants, representing nearly 10% increase from the Unified Human Gastrointestinal Genome (Kim et al., 2021).

The commonly used intestinal microbial analysis methods mainly involve deep sequencing of the variable region of bacterial 16S rRNA gene and metagenomic analysis. Both 16S rRNA gene sequencing and metagenomic analysis revealed that the human intestine is mainly composed of the common core bacteria from two phyla, namely Firmicutes and Bacteroides, whereas the remaining intestinal microbiota is highly diverse (Ghazalpour et al., 2016). This diversity often involves less abundant representation from the phyla Proteobacteria, Verrucomicrobia, Actinobacteria, Fusobacteria and Cyanobacteria and from the domain Archaea. However, Jin et al. (2022) recently designed a Hi‐seq‐PacBio hybrid, ultra‐deep metagenomic sequencing method, which is superior to the existing methods for genomic and functional characterization of low‐abundance and ultra‐low‐abundance species in the human gut bacterial community. Culture‐based methods also play a crucial role in the study of intestinal microbes. Bellali et al. (2021) isolated 495 bacterial strains by using the culture group method. However, culturing strains from the intestinal tract is challenging because of the limiting culture conditions and the low abundance of such strains or the death and damage of bacteria in the distal intestinal tract. Aranda‐Díaz et al. (2022) generated hundreds of in vitro communities cultured from diverse stool samples in various media; certain media generally preserved the inoculum composition. Upon colonization of germ‐free mice, the community composition was maintained, and the host proteome resembled that of the host from which the community was derived.

## INTESTINAL MICROBES INVOLVED IN LIPID METABOLISM IN THE HOST

Microbiome can act as a regulator of lipid metabolism in the intestine by regulating the processes such as digestion, absorption, storage and secretion of dietary lipids. Therefore, the intestinal microbiome can help increase the amount of energy obtained from the diet; regulation of signalling pathways in a host may affect its energy balance (Ghazalpour et al., 2016). Microbial suppression of fasting induced adipocyte protein factor (*Fiaf*) in the intestinal epithelium results in reduced levels of the circulating lipoprotein lipase (LPL), which is an inhibitor and the key protein for triglyceride metabolism, increased LPL activity in adipocytes and enhanced storage of liver‐derived triacylglycerols in fat cells (Kobyliak et al., 2016). Intestinal micro‐organisms inhibit *fiaf* gene expression and increase lipid storage in white adipose tissue (WAT). GF mice showed increased expression of WAT *fiaf*, an inhibitor of LPL activity, in the small intestine compared with conventional mice (Davis *et al*. 2016). After the microbial community is regularized, *fiaf* expression is decreased and the LPL activity is increased, which lead to WAT lipid storage (Paul et al., 2016). In a study, researchers transferred faeces from twins with different obesity phenotypes to microbiome‐free mice and found that the mice acquired the phenotypes from human donors, thereby confirming the critical role of the human gut microbiome in lipid metabolism regulation (Ridaura et al., 2013). In addition, alteration of the gut microbiome reduced fat mass and body weight in rodents and improved insulin sensitivity (Rastelli et al., 2018). Ley et al. (2006) demonstrated that transferring the gut microbiome of mice with genetically induced obesity (i.e. ob/ob mice) to GF mice led to the elevated fat mass and body weight.

*Bacteroides* are well‐known as ‘lean microbes’, whereas *Faecalibacterium*, Lachnospiraceae, Ruminococcaceae and *Anaerofilum*, belonging to the phylum Firmicutes, are known as ‘obese microbes’ (Mariat et al., 2009). Ley et al. (2005) reported that the abundance of Bacteroidetes in patients with obesity was less and that of Firmicutes was more than those in the lean controls. In lean individuals, reduction in the abundance of Firmicutes relative to that of Bacteroidetes was associated with faecal calorie loss. Increased Bacteroidetes abundance was associated with weight loss but not with changes in the dietary calorie content over time (Ley et al., 2006). Bacteroidetes play a key role in decomposing plant starches and fibres to provide energy, which might partly explain the mechanism of fat deposition reduction by Bacteroidetes (Ley et al., 2005). Therefore, predisposition to increased body fat or obesity is determined by the Firmicutes:Bacteroidetes (F:B) ratio (Dreyer & Liebl, 2018). However, some meta‐analyses have found no significant association between this ratio and BMI or obesity, suggesting that this ratio or specific microbial characteristics do not vary between normal and obese human intestinal microbiota (Sze & Schloss, 2016). Transplantation of the intestinal microbiota of lean individuals into obese recipients resulted in no significant changes in weight or BMI; however, insulin sensitivity and other markers of lean body weight improved in obese individuals (Allegretti et al., 2020). It is generally believed that increased Firmicutes abundance is associated with a high obesity risk, though some other studies have attributed the obesity risk to the decreased abundances of Actinomycetes (*Bifidobacterium*) or Verrucomicrobia (*Akkermansia muciniphila*) rather than the F:B ratios (Amabebe et al., 2020). Many studies have shown that low gut microbial diversity and richness are associated with an increased risk of obesity. However, Kasai et al. (2015) reported that 33 patients with obesity in Japan had significantly higher bacterial diversity than 23 nonobese individuals. These differences may be attributed to interpersonal differences, insufficient sample size and different methods.

*Lactobacillus paracasei* inhibits lipid oxidation to promote lipid storage and *Escherichia coli* promotes lipid oxidation (Araújo et al., 2020; Lamichhane et al., 2021). *Clostridium bifermentans* or its metabolites may be the key microbial factors affecting lipid absorption (Kleerebezem, 2018). An increase in *A. muciniphila* abundance leads to a significant reduction in faecal heat (Basolo et al., 2020). In addition, compared with that in conventional animals, the absorption of lipid in the duodenum and jejunum of germ‐free animals was significantly reduced, and the absorption of lipid in different organs was slower. Therefore, it is not surprising that bacterial dysregulation is associated with numerous metabolic diseases, such as malnutrition or undernutrition (Vonaesch et al., 2018) and obesity. In an animal study, among the intestinal micro‐organisms of chickens, the abundance of Fusobacteria in the obese group (8%) was found to be significantly lower than that in the lean group (18%). Conversely, the proportion of Proteobacteria in the obese group was 33% and that in the lean group was approximately 24% (Ding et al., 2016). The fat deposition in chickens with the lowest *Methanobrevibacter* abundance was significantly lower than that in chickens with the highest *Methanobrevibacter* abundance, and no difference was observed in body weight between the two groups. Additionally, 20% of chickens with the highest abundance of *Mucispirillum schaedleri* had significantly lower fat content than the 20% of chickens with the lowest *M. schaedleri* abundance (Wen et al., 2019). Notably, the intestinal fungal community plays a key role in ensuring long‐term stability of the intestinal ecosystem and in regulating host lipid metabolism. For instance, Saccharomycetales spp. interacts with intestinal bacteria and are positively related to SCFA producers to influence insulin sensitivity (Dalamaga et al., 2022). These results suggest that comprehensive cross‐boundary analyses may broaden our horizons and help us in identifying new prevention and treatment targets for metabolic disorders.

## INTESTINAL MICROBIAL METABOLITES REGULATING LIPID METABOLISM

### Short‐chain fatty acids

**FIGURE 1:**
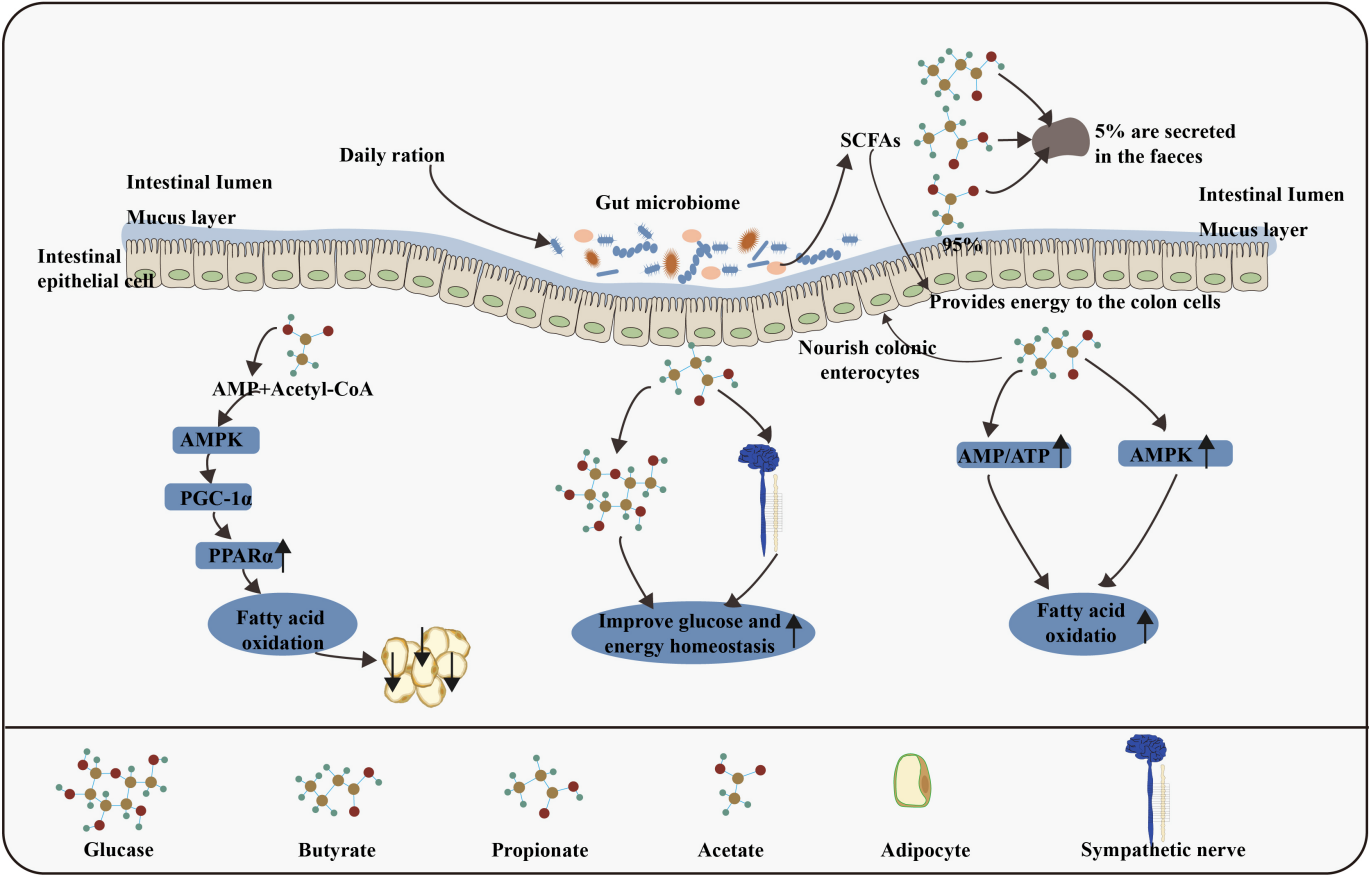
Relationship between SCFAs and lipid metabolism in animals. The contents of acetate, propionate and butyrSate in SCFAs were the highest (≥95%). Acetate inhibits chylomicron secretion by enterocytes but promotes lipid oxidation by a mechanism involving acetate absorption by enterocytes, its metabolism to acetyl‐CoA and AMP and the subsequent up‐regulation of the AMPK/PGC‐1α/PPARα pathway. Propionate and butyrate might improve glucose and energy homeostasis by inducing intestinal gluconeogenesis and sympathetic nerve activity. Moreover, acetate and butyrate might directly increase hepatic AMPK phosphorylation and activity via an increase in the ratio of AMP to ATP and the up‐regulation of PPARα target genes, thereby increasing fatty acid oxidation and glycogen storage. AMP, adenosine monophosphate; AMPK, adenosine monophosphate‐activated protein kinase; PGC‐1α, peroxisome proliferator‐activated receptor γ coactivator‐1α; PPARα, peroxisome proliferator‐activated receptor α; ATP, adenosine triphosphate.

Short‐chain fatty acid production is one of the main mechanisms through which intestinal micro‐organisms affect host global lipid metabolism. SCFAs are produced by the fermentation of indigestible food by intestinal micro‐organisms and include acetate, propionate, isobutyrate, butyrate, isovalerate and valerate. The molar ratio of acetate, propionate and butyrate in the colon and faeces was reported to be approximately 3:1:1 (Besten et al., 2013). The total concentration of SCFAs decreased from 70–140 mM in the proximal colon to 20–70 mM in the distal colon, depending on the diet. In the caecum and large intestine, 95% of the SCFAs produced are rapidly absorbed by colon cells, whereas the remaining 5% are secreted in the faeces (Al‐Lahham et al., 2012). These SCFAs serve as both signalling molecules in the body and important energy sources as they fulfil approximately 10% of the human body's caloric requirements for optimal functions. SCFAs can also control body weight by regulating energy intake, energy acquisition, host energy and substrate metabolism, which indicate their critical role in the pathophysiology of obesity and related diseases (Figure 1). In the liver, up to 70% of acetate is absorbed and used as: an energy source, a substrate for the synthesis of cholesterol and long‐chain fatty acids and a common substrate for the synthesis of glutamine and glutamic acid. After rapid absorption by the intestinal tract, SCFAs not only store energy, but also reduce osmotic pressure and play an important role in maintaining normal functioning of the large intestine and morphology and function of the colonic epithelial cells (Al‐Lahham et al., 2010). Studies have reported that these SCFAs are linked to insulin sensitivity and exert organogenic effects on adipose tissue function, lipid storage capacity and liver and skeletal muscle substrate metabolism (Morrison & Preston, 2016). Excess SCFAs that are not absorbed or metabolized by the intestinal epithelial cells are transported to the liver via hepatic veins, where they serve as precursors for gluconeogenesis, lipogenesis and cholesterolgenesis (Topping & Clifton, 2001).

Acetate and its metabolic intermediates, especially AMP, have been reported to activate AMPK, which is a metabolic sensor regulating fatty acid, and induce cholesterol metabolism to regulate lipid metabolism (Araújo et al., 2020). Acetate decreases lipid accumulation and secretion, leading to increased lipid consumption in the intestinal tract. Propionate is a precursor of gluconeogenesis in the liver, and according to estimates, humans use 50% propionate as a substrate for hepatic gluconeogenesis (Roy et al., 2006). Propionate increases the risk of diabetes; the higher the propionic acid content in faeces, the higher is the risk of T2D (Sanna et al., 2019). Butyrate is absorbed and acts, through monocarboxylic acid transporter 1 and solute transporter solute carrier family 5 member 8 on colon cells, as a histone deacetylase inhibitor or GPR signalling molecule to perform its function (Cresci et al., 2010). Degradation of 4‐aminobutyric acid (the degradation product of butyric acid) is associated with increased insulin secretion, which improves the body's insulin response (Sanna et al., 2019). A study in mice revealed that dietary butyrate supplementation prevented and reversed high‐fat‐diet‐induced obesity by down‐regulating the expression and activity of peroxisome proliferator‐activated receptor γ (PPARγ), promoting a change from lipogenesis to lipid oxidation (Den Besten et al., 2015). Additionally, a small amount of propionate and butyrate and a large amount of acetate in circulation can directly affect the metabolism and function of the peripheral adipose tissue, liver and muscle substrates (Araújo et al., 2020). Propionate administration alone has been shown to reduce the lipid content in overweight adult hepatocytes (Chambers et al., 2015). In the distal intestine, micro‐organisms ferment dietary polysaccharides to produce monosaccharides and SCFAs, whose subsequent absorption stimulates triglyceride re‐synthesis in the liver.

Both acetate and propionate might inhibit intracellular lipolysis; this effect can reduce lipid overflow and heterotopic accumulation of fat, thus improving insulin sensitivity. Acetate and butyrate might increase oxidation of local muscle fat in the AMPK‐dependent manner or by changing the oxidation state of muscle fibre, thereby increasing the use and conversion capacity between major fuel lipids and carbohydrates (Canfora et al., 2015). Acetate produced by *E. coli* RB01 can reduce the content of intracellular triglycerides and cholesterol ester and lipid droplet size, thus inhibiting the bacteria's ability to increase the consumption of intestinal lipid. Acetate also decreased lipid accumulation and secretion, resulting in increased lipid consumption in the intestinal tract (Araújo et al., 2020). Acetate seems to be the main effector for regulating lipid metabolism in *E. coli*. SCFAs probably enhance the intestinal barrier function, which further supports their anti‐inflammatory potential. In several studies using intestinal cell lines, SCFAs (particularly butyrate) were found to improve epithelial barrier function and intestinal permeability by modulating the expression of tight junction protein and mucins (Elamin et al., 2013). Butyrate can regulate obesity‐induced chronic low‐grade inflammation by activating anti‐inflammatory T_REG_ cells and suppressing pathways involved in proinflammatory cytokine and chemokine production (Canfora et al., 2015).

In addition, some intestinal microbial species, mainly bacilli, carry a specialized branched‐chain ketone acid dehydrogenase complex, which generates energy from the oxidative form of branched‐chain amino acids directly. This also leads to the production of branched‐chain fatty acids (BCFAs) (Portune et al., 2016). Previous studies have shown that BCFAs, similar to SCFAs, regulate glucose and lipid metabolism in the liver (Heimann et al., 2016). However, studies are warranted to confirm the effects of BCFAs on lipid metabolism in the host.

**FIGURE 2:**
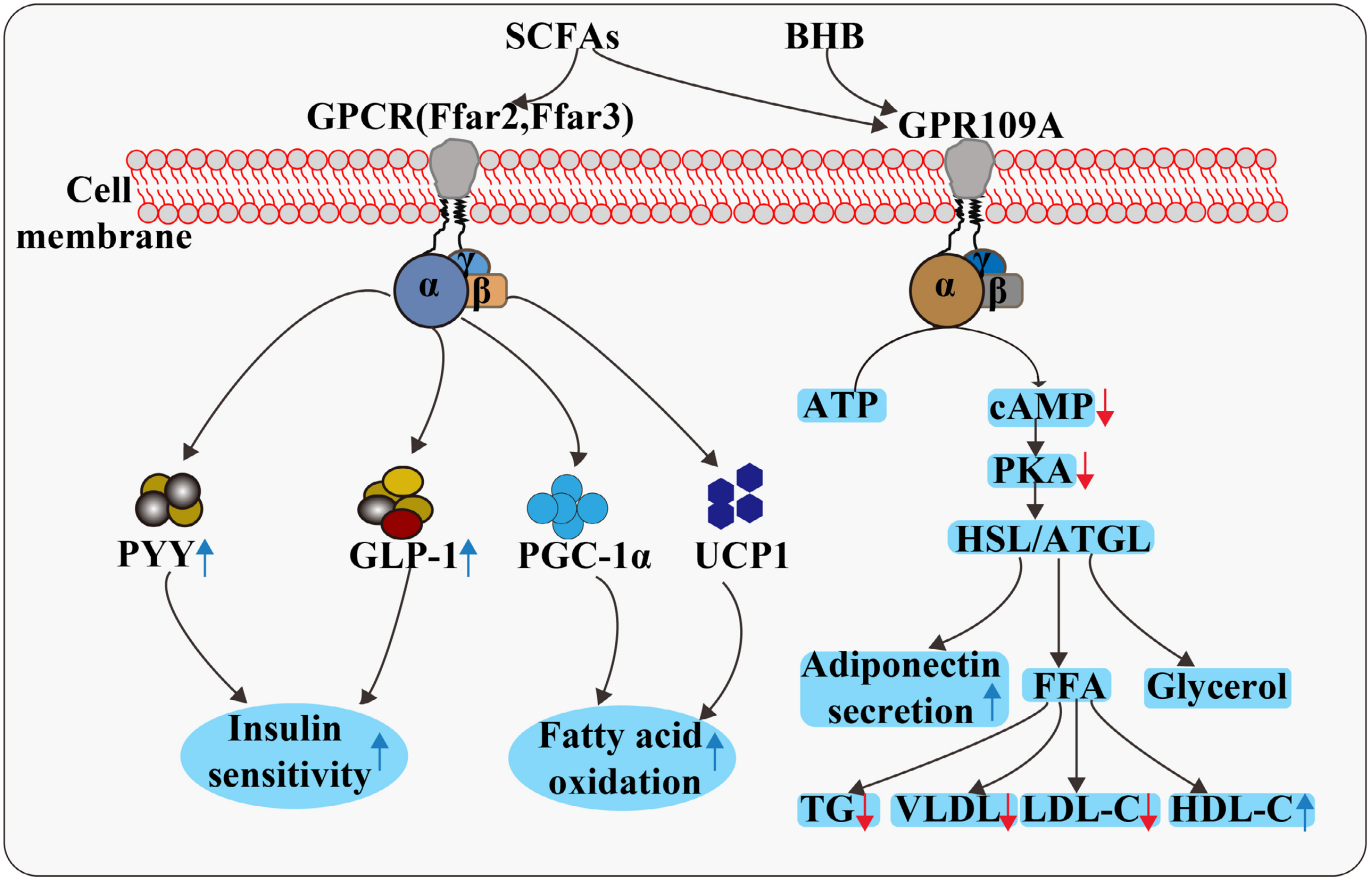
Mechanism of action of GPCRs. SCFAs bind to GPR41 and GPR43 in the distal intestine to produce the intestinal hormones, namely PYY and GLP‐1, which affect satiety and glucose homeostasis. In brown adipocytes, SCFAs directly promote the expression of peroxisome PGC‐1α and UCP1 through a GPR43‐independent pathway, thus increasing fatty acid oxidation. Activation of GPR109A in the adipocyte results in inhibition of adenylate cyclase activity and subsequent reduction in cAMP levels and PKA and HSL/ATGL activity. This results in reduced hydrolysis of TG and subsequent suppression of FFA and glycerol release from the adipocytes. At the same time, adipocytic secretion of adiponectin is increased. The reduction in substrate availability to the liver limits TG and VLDL synthesis and subsequently reduces serum concentration of TG and LDL‐C and increases that of HDL‐C. GPCR, G‐protein‐coupled receptor; PYY, peptide YY; GLP‐1, glucagon‐like peptide‐1; PGC‐1α, peroxisome proliferator‐activated receptor γ coactivator‐1α; UCP1, uncoupling protein 1; ATP, adenosine triphosphate; cAMP, cyclic adenosine monophosphate; PKA, protein kinase A; HSL, hormone sensitive lipase; ATGL, adipose triglyceride lipase; TG, triglyceride; FFA, free fatty acid; VLDL, very low‐density lipoprotein; LDL‐C, low‐density lipoprotein cholesterol; HDL‐C, high‐density lipoprotein cholesterol.

SCFAs exert significant effects on energy expenditure and insulin sensitivity in surrounding metabolic tissues through different G‐protein‐coupled receptors (GPCRs) such as Ffar2 and Ffar3 (GPR43 and GPR41) (Figure 2). Due to differences in chain lengths, Ffar2 and Ffar3 exhibit different activities for SCFAs; activity relationship for Ffar2 is: acetate = propionate > butyrate, whereas that for Ffar3 is: butyrate = propionate > acetate (Brown et al., 2003). Both these receptors are expressed in the human WAT, skeletal muscles and liver (Al‐Lahham et al., 2010), suggesting that SCFAs may also directly affect substrate and energy metabolism in peripheral tissues. These SCFAs bind to GPR41 and GPR43 in the distal intestine and participate in the regulation of lipid metabolism (Brown et al., 2003).

GPR43, the key receptor for lipid metabolism regulation by SCFAs, is mainly activated by acetate and propionate, followed by butyrate (Kasubuchi et al., 2015). GPR43 is highly expressed in mammalian white adipocytes, but barely expressed in black adipocytes, muscles and the liver (Kimura et al., 2013). GPR43 activated by SCFAs could couple to G_i/o_‐PLC‐PKC signalling in white adipocytes, this reduces insulin sensitivity in white adipocytes through inhibition of AKT phosphorylation, finally reducing fat storage in white adipocytes. In addition, GPR43 could couple to G_i/o_‐cAMP‐PKA signalling in white adipocytes, this inhibits the activity of triglyceride decomposition‐related enzymes such as hormone‐sensitive lipase and adipose triglyceride lipase, thereby reducing lipolysis in white adipocytes (Ohira et al., 2013). However, in the liver and muscle, SCFAs could regulate fatty acid metabolism through both GPR43‐independent and GPR43‐dependent pathways.

GPR109A also regulates lipid metabolism and is demonstrated to be a receptor of BHB; its activation leads to anti‐lipolysis effects (Figure 2). BHB is a ligand of GPR109A that inhibits lipolysis and exerts anti‐inflammatory effects on cells (Lee et al., 2020). GPR109A is a seven‐transmembrane GPR belonging to the Gαi family, which is expressed in the adipocytes of white and brown adipose tissues, keratinocytes, immune cells and epithelial cells of the colon and retina. A recent study reported that GPR109A is expressed in the normal human and mouse livers (Jadeja et al., 2019). The receptor when activated in response to changes in the metabolic and/or immune status has been characterized as a metabolic sensor, modulating cell signalling, which is coupled to energy and lipid metabolism, as well as immune cell function, both directly and indirectly. Jadeja et al. (2019) reported quantifiably significant differences in the amount of weight gained and detected related fat accumulation in GPR109A knockout mice compared with age‐ and gender‐matched wild‐type mice, despite similarities in food intake between the two groups. Moreover, the morphological and histological analyses of adipose tissue revealed an inverse relationship among the expression of key lipogenic genes, adipocyte hypertrophy and GPR109A expression.

### Biliary saline hydrolyase

Biliary saline hydrolyase secreted by the intestinal microbiota could affect lipid metabolism in mammals by converting conjugated bile acids into free bile acids (Liang et al., 2020). Various mammalian intestinal bacteria could produce BSH, including probiotic bacteria (such as *Lactobacillus* and *Bifidobacterium*) and pathogenic bacteria (such as *Enterococcus*, *Streptococcus*, *Bacteroides*, *Brucella abortus*, *Listeria monocytogenes* and *Clostridium perfringens*) (Dong & Lee, 2018). Archaea species in the human intestine such as *Methanosphaera stadtmanae* and *Methanobrevibacter smithii* are reported to produce BSH (Jones et al., 2008). Similar to probiotic bacteria, some strains of yeasts can produce the enzyme BSH.

Bile acids are amphiphilic molecules and hence can promote the emulsification of intestinal lipids and their digestive products, thereby promoting the digestion and absorption of lipids (González‐Regueiro et al., 2017) and inhibiting the precipitation of cholesterol in bile. The solubility of free bile acids decreases compared with that of unconjugated bile acids, which decreases the intestinal reabsorption of bile acids, and only approximately 95% of bile acids return to the liver through enterohepatic circulation. To make up for the loss of bile acids caused by BSH, the feedback inhibition of bile acid synthesis reduces and the conversion of cholesterol to bile acids increases (Park et al., 2008). This route of de novo bile acid biosynthesis in the liver maintains the pool size and is crucial for cholesterol turnover in humans and most other mammals, resulting in a decrease in the serum cholesterol levels (Reis et al., 2017). In addition, because the dissolution and absorption efficiency of free bile acids is not as good as that of conjugated bile acids, the hydrolysis of conjugated bile acids by BSH would lead to lipid malabsorption, eventually leading to a reduction in triglyceride levels in the liver and serum and causing weight loss in the host (Wang et al., 2019). Furthermore, cholesterol may co‐precipitate with free bile acids and be excreted from faeces (Tsai et al., 2014), which can further reduce the serum cholesterol levels. These biological activities of BSH can promote reduction in the human serum cholesterol level. The strain expressing this enzyme could maintain human health and alleviate diseases in patients with high cholesterol (Wang et al., 2012), including those with cardiovascular disease. In animal studies, the effect of BSH in birds has been reported to be the same as that in mammals. Geng et al. (2020) reported that the three BSH inhibitors increased the serum total cholesterol and very‐low‐density lipoprotein levels, body weight gain and feed efficiency in chickens.

In conclusion, various intestinal bacteria synthesize and secrete BSH, which could enhance catabolism of cholesterol in the liver, increase excretion of cholesterol in bile and down‐regulate the absorption efficiency of lipids in the intestine, resulting in decrease in the triglyceride and cholesterol levels in the liver and serum.

### Trimethylamine‐*N*‐oxide

In mammals, intestinal microbiota could use their own unique trimethylaminelyase to catalyse the cleavage of C–N bonds in nutrition such as choline and carnitine to produce trimethylamine (TMA). TMA is absorbed in the intestine and further oxidized to TMAO by flavin‐containing monooxygenases (FMOs) (mainly FMO3) in the liver before entering the bloodstream (Koeth et al., 2013). FMO3 is identified as a powerful modifier of cholesterol metabolism and RCT, and its deficiency leads to decreased glucose and lipid levels in the circulation and liver, whereas FMO3 overexpression has opposite effects (Shih et al., 2015). TMAO could inhibit RCT by inhibiting the expression of CYP7A1, sterol 27 α‐hydroxylase, these two rate‐limiting enzymes in bile acid synthesis, and bile acid transporter in the liver, which would eventually induce cholesterol accumulation in peripheral cells and their conversion to foam cells (Koeth et al., 2013). Many studies have reported these effects of TMAO on lipid metabolism. For example, Gao et al. (2014) reported that TMAO increased the levels of total cholesterol and triglyceride in the liver of mice, but decreased the levels of total cholesterol and triglyceride in plasma. Moreover, Koeth et al. (2013) reported that the diet supplemented with l‐carnitine aggravated the degree of atherosclerosis in mice, and the direct addition of TMAO to the diet reduced the plasma cholesterol in mice. Because of these adverse effects of TMAO, controlling the contents of nutrients such as choline and carnitine in mammalian diets is necessary.

### Lipopolysaccharide

Studies have shown that intestinal microbiota can activate the inflammatory state of obesity through the activity of LPS. LPS is a cell wall component of Gram‐negative bacteria that detaches after death of bacteria and is absorbed by intestinal capillaries. A high‐fat diet could increase the proportion of Gram‐negative bacteria in the intestines of mammals, resulting in an increase in the plasma LPS concentration (Clarke et al., 2012). LPS could induce an increase in plasma free fatty acid levels and liver triglyceride levels, but decreased the plasma triglyceride levels (Guo et al., 2016).

**FIGURE 3:**
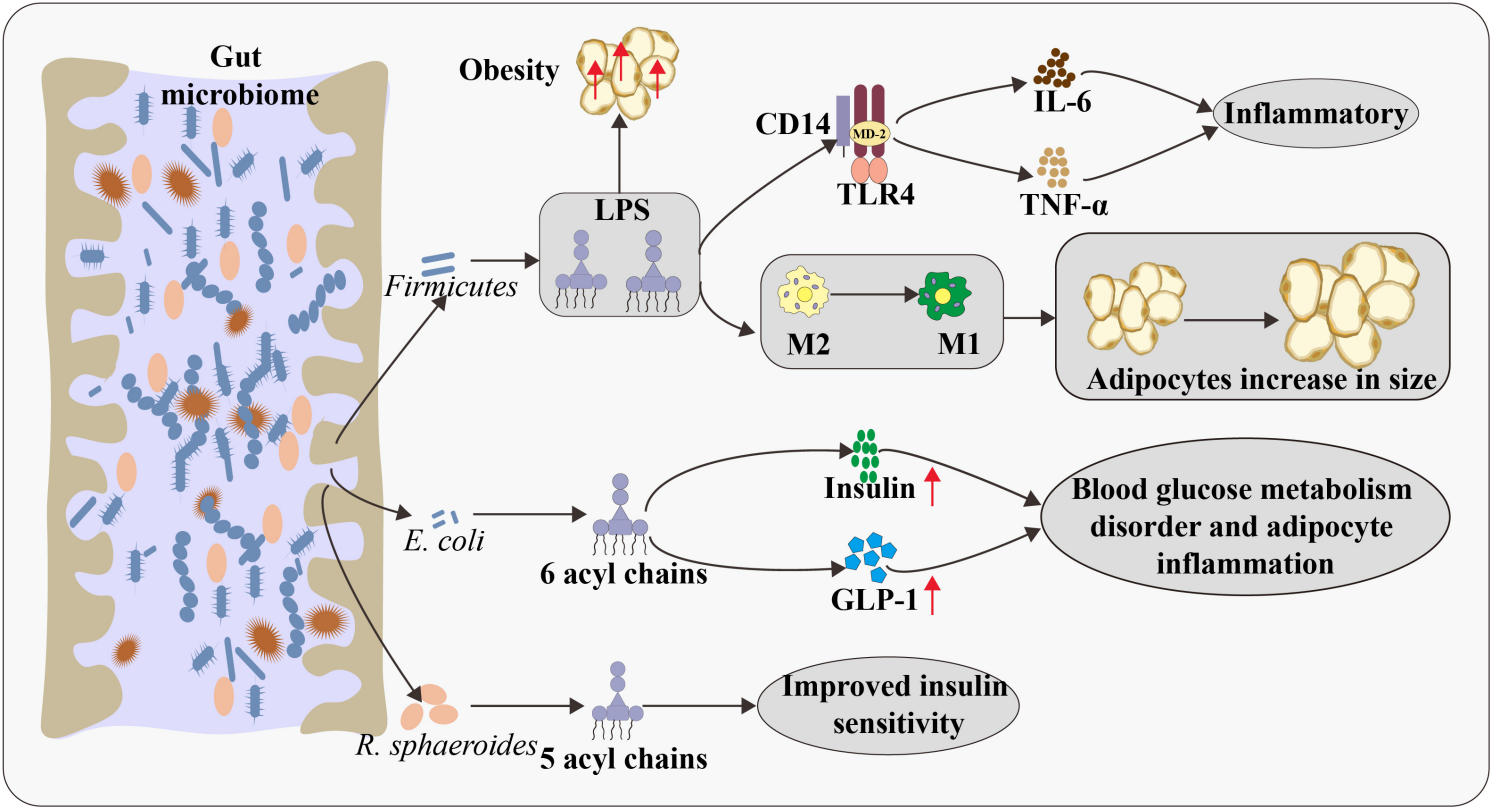
Relationship between LPS and lipid metabolism in animals. Firmicutes can produce LPS, which could increase visceral and subcutaneous depots in mammals and eventually lead to obesity. LPS can trigger the inflammatory process by binding to the CD14 TLR4 complex in the gut wall. LPS is involved in the transition of macrophages with the M2 phenotype to the M1 phenotype. In lean VAT, the macrophages are predominately of the M2 phenotype. During transition towards obesity, adipocytes increase in size, the phenotype of macrophages changes to the inflammatory M1 phenotype and an increasing number of large adipocytes die of pyroptosis. LPS, lipopolysaccharide; TLR4, toll‐like receptor‐4; M, macrophages; VAT, visceral adipose tissue; GLP‐1, glucagon‐like peptide‐1.

In mammals, the abundance of Firmicutes in the guts of obese individuals is higher than that in normal individuals, though the abundance of *Bacteroides* is less. Firmicutes are Gram‐negative bacteria that produce LPS (Clarke et al., 2012), this could increase visceral and subcutaneous depots in mammals, eventually leading to obesity (Figure 3). Another metabolic disease associated with LPS is NAFLD. In the liver, LPS increases the expression of tumour necrosis factor‐α (TNF‐α) and induces the release of chemokines (Henao‐Mejia et al., 2012), which cause hepatocyte injury and steatosis by promoting neutrophil infiltration, finally leading to NAFLD (Guo et al., 2016). Obesity and insulin resistance are associated with low‐grade chronic systemic inflammation (Wellen & Hotamisligil, 2005). We identified bacterial LPS as an inflammatory factor that triggers the onset of insulin resistance, obesity and diabetes. LPS‐treated mice exhibited inflammation, as revealed by the increased expression of genes coding for cytokines IL‐6, TNF‐α, IL‐1 and PAI‐1 in the adipose depots, liver and muscles (Cani et al., 2007). The level of serum LPS is almost doubled in obese, diabetic or high‐fat‐fed individuals due to processes involving an increase in chylomicron formation and decrease in intestinal barrier integrity and activity of the enzyme alkaline phosphatase, which is responsible for the cleavage of LPS in the intestine (Ghanim et al., 2009). In addition, genetically obese fa/fa rats and ob/ob mice quickly develop steatohepatitis after exposure to low doses of LPS (Vyberg et al., 1987). However, polymyxin B treatment, which specifically eliminates Gram‐negative bacteria and further quenches LPS, diminishes hepatic steatosis (Pappo et al., 1991). In general, endotoxaemia caused by bacterial LPS is believed to cause inflammation and impair metabolic health. However, a recent study suggested that LPS from different bacteria can have different effects on host health, and some may even be beneficial (Anhê et al., 2021). LPS of *E. coli* (six acyl chains) can damage intestinal barrier function, delay intestinal glucose absorption, increase insulin and GLP‐1 secretion and promote blood glucose metabolism disorder and adipocyte inflammation. LPS (five acyl chains) of *Rhodobacter sphaeroides* was reported to be metabolically beneficial, counteracting the harmful effects of *E. coli* LPS and improving insulin sensitivity in obese mice (Figure 3).

Studies on the effects of LPS on poultry are limited compared with relevant studies on mammals. Nevertheless, some effects such as hyperthermia, inflammation and lethargy have been reported in chickens (Xie et al., 2000). Moreover, LPS could affect lipid metabolism in birds, similar to that in mammals. Rauber et al. (2012) observed that LPS (from *Salmonella typhimurium*) could induce the increase in total cholesterol in plasma in broiler chickens; however, LPS treatment did not affect the plasma triglycerides levels.

## OTHER INFLUENCING FACTORS

### Diet

The intestinal microbiota responds rapidly to dietary changes, and the microbial composition undergoes changes rapidly at the species and family levels within 24–48 h of the dietary intervention (Sonnenburg & Bäckhed, 2016). Studies have proved that controlling the feeding behaviour, including the time, duration and frequency of feeding, may affect the composition and function of intestinal micro‐organisms and even the health of the host (Kaczmarek et al., 2017). A study on the effects of human mealtimes on the intestinal and oral microbiota reported that the timing of meals affects the circadian rhythms of salivary microbes, with late meal timings increasing salivary groups commonly thought to be proinflammatory, affecting body weight, cortisol rhythm, basal metabolic rate and glucose tolerance (Collado et al., 2018). Studies have shown that fructose ingested in large doses is converted into acetic acid by intestinal microbes, which is then converted into acetyl‐CoA by ACSS2 (acyl‐CoA synthetase short‐chain family member 2) in the liver to promote the production of fatty acids (Zhao et al., 2020). These diet‐induced microbial changes are transient, and they disappear after discontinuation of the diet, suggesting a possible need for continuous substrate intake. Therefore, sustainable dietary changes are important to maintain the effects of diet on the intestinal microbiome composition. A study investigating the effects of inulin‐type fructan on obese women reported changes in the microbial profile, alteration of lipid metabolism and a reduction in fat mass and serum LPS; some metabolites such as phosphatidylcholine, lactate and hippurate were identified (Choque et al., 2018). Furthermore, fibre, magnesium, biotin and vitamin E have all been shown to impact visceral fat mass accumulation mediated by the intestinal microbiota (Le Roy et al., 2020).

A positive correlation between dietary diversity and microbial stability was observed in a longitudinal observational study by Johnson et al. (2019). Nevertheless, dietary patterns that consider dietary quality and diversity may provide information on persistent microbial shifts for future habitual eating strategies. For example, Mediterranean and low‐fat dietary patterns were shown to partially restore keystone taxa loss in 33 participants with obesity and varying levels of metabolic dysfunction (Haro et al., 2017). Dao et al. (2016)evaluated the association between *A. muciniphila* abundance and gene richness of the host's faecal sample, as well as between diet and bioclinical parameters. The results indicated that people with greater gene richness and greater abundance of *A. muciniphila* displayed the healthiest metabolic status, particularly regarding fasting plasma glucose, plasma triglycerides and distribution of body fat.

### Probiotics, prebiotics and synbiotics

Probiotics are defined as living microbes that can provide health benefits to the host when ingested in sufficient quantities (Li et al., 2021). They are also involved in lipid metabolism of the host and induce weight loss. Next‐generation probiotics such as *Faecalibacterium prausnitzii*, *A. muciniphila* or *Clostridia* strains have been demonstrated to be present in the microbiota of a majority of people. Their reduction has been related to the increased risks of obesity and T2DM (Wang et al., 2019). The effect of *Lactobacillus*, *Bifidobacterium* and *Saccharomyces* on weight loss and/or fat deposition in overweight adults has been reported. For example, weight loss is enhanced by *L. gasseri* and *L. amylovorus*; hypocaloric diet comprises *L. plantarum* and *L. rhamnosus* (Amabebe et al., 2020). Kadooka et al. (2010) found that overweight humans who consumed fermented milk containing *L. gasseri* experienced significant reduction in abdominal visceral and subcutaneous fat, body weight, BMI and waist and hip circumference compared with those who consumed unsupplemented fermented milk. In addition, Cani and Everard (2014) reported a negative correlation between *A. muciniphila* abundance and overweight, obesity, untreated T2DM or hypertension; Chaiyasut et al. (2022) found that the supplementation of probiotic mix (*L. paracasei* HII01, *Bifidobacterium breve* and *Bifidobacterium longum*) improved the intestinal barrier function, lipid profile and obesity‐related biomarkers in humans. Regarding the effects of probiotics, most studies have demonstrated a decrease in body weight and BMI among participants aged >18 years (Vallianou et al., 2020). More notably, *L. gasseri* BNR17 has been approved by the Korean Food and Drug Administration as a functional ingredient for reducing body fat (Jung et al., 2013). In an animal study, *A. muciniphila* was found to be inversely related to the onset of inflammation, altered adipose tissue metabolism and metabolic disorders in obese rodents (Schneeberger et al., 2015). Furthermore, yeast probiotics such as *Saccharomyces boulardii* were shown to improve the microbiota metabolic profiles of genetically obese and diabetic mice, with reduced Firmicutes abundance and increased Bacteroidetes abundance along with a corresponding decrease in fat mass (Osterberg et al., 2015). VSL#3 promotes epithelial tight junction integrity and anti‐inflammation, thereby preventing fat accumulation and weight gain (Hu et al., 2015). Most studies have reported that probiotics are beneficial to host health; however, a few studies have reported no health benefit or even obesogenic effects associated with probiotics. For instance, large weight gain observed in livestock that were fed huge amounts of probiotics raises speculations that obesity in humans may be associated with high consumption of foods enriched with probiotics. Therefore, more comprehensive randomized controlled trials are required to ascertain the efficacy of probiotics in the treatment and prevention of obesity locally or globally.

Prebiotics are the selectively fermented nondigestible food ingredients that cause specific changes in the composition and/or activity of the gastrointestinal microbiota, thereby conferring health benefits to host (Bindels et al., 2015). Accumulating evidence indicates that the consumption of diets rich in prebiotics is associated with reductions in food intake, body fat content and body weight gain, especially in overweight and obese individuals (Roberfroid et al., 2010). Oligosaccharides (e.g. inulin), galactose oligosaccharides, can increase satiation by up‐regulating GLP‐1 and PYY expressions and reduce ghrelin secretion, thereby reducing the intake (Cani et al., 2009). In a study, administration of oligofructose (21 g/day) for 12 weeks resulted in weight loss, decreased ghrelin expression, increase in PYY levels, low calorie intake and low plasma glucose and insulin levels in 48 adults (Cani et al., 2009). Some animal studies found that cyanidin 3‐glucoside (C3G) supplementation to genetically obese mice for 16 weeks resulted in decreases in weight gain, improved glucose homeostasis and hepatic steatosis, ameliorated tolerance to cold exposure, stimulated brown adipose tissue activity and browning of subcutaneous WAT. Most meta‐analyses have found only minor or no changes in body weight and BMI as well as minor improvements in serum C‐reactive protein, total cholesterol and LDL‐cholesterol levels with the administration of prebiotics (Vallianou et al., 2020). Tarantino and Finelli (2015) recommended the use of probiotics in patients with different stages of NAFLD and T2D as the adjunct to standard treatment regimens because they decrease the low‐grade systemic inflammatory response; probiotics treatment may reduce the liver fat and aspartate aminotransferase level in nonalcoholic steatohepatitis; patients with NAFLD exhibited increased liver aminotransferase levels, and in patients with NAFLD and atherogenic dyslipidaemia, a combination therapy of statins and probiotics is reported to be more effective than monotherapy in lowering cholesterol and the products of intestinal flora metabolism. Although the use of probiotics and prebiotics has been recommended in the treatment and prevention of patients with obesity‐related NAFLD, their therapeutic use is not supported by high‐quality clinical studies. Therefore, we should consider the influence of the heterogeneity in genotype, lifestyle, diet and the complex aetiology of obesity and its associated metabolic disorders on patients with NAFLD.

Intestinal microbiota are the resident probiotic bacteria in the gastrointestinal tract that can be introduced as a plausible regulator of IL‐17A production and functions. They are the main inducer of Th17 differentiation, which is the main source of IL‐17A. Additionally, interaction of SCFAs with GPR43 in the immune cells leads to the modulation of IL‐17A expression (Masui *et al*., 2013). For instance, Fernando et al. (2016) revealed that butyrate down‐regulates the expression of IL‐17A pathologic levels. In addition to protecting against pathogenic agents, the cytokine IL‐17A also participates in alleviating metabolic and proinflammatory diseases. An investigation demonstrated that segmented filamentous bacteria (SFB) accelerate NAFLD progression in the IL‐17A‐dependent manner (Harley et al., 2014). Intestinal microbiota modulate immune responses in proinflammatory diseases through several mechanisms including down‐regulation of IL‐17A and alteration of the intestinal microbiota population. For instance, up‐regulation of SFB may be associated with proinflammatory diseases (Douzandeh‐Mobarrez *et al*., 2019). This major cytokine is closely associated with atherosclerosis, another major co‐morbidity of obesity. Results from animal studies suggest that IL‐17 plays a proatherogenic role (Iwakura *et al*., 2011), but exerts an atheroprotective effect in humans through the cross‐regulation of IFN‐γ‐producing Th1 cells (Danzaki *et al*. 2012). Accordingly, Simon *et al*. (2013) found an inverse association between the levels of circulating IL‐17 and the risk of major cardiovascular events. Tarantino *et al*. (2014) confirmed an indirect role of IL‐17 in the early diagnosis of subclinical atherosclerosis in obese patients, in the sense that its circulating levels, strongly linked to those of eotaxin, could induce the allergic–hyperergic mechanism, eventually triggering the disease (Tarantino *et al*., 2014).

Mixtures comprising live micro‐organisms and substrate(s) and selectively utilized by host micro‐organisms that confer health benefit to the host are called synbiotics (Swanson et al., 2020). Diet supplementation with synbiotics by using strictly selected strains, such as *L. gasseri* strains that have been documented to exhibit weight reduction and ant‐inflammatory properties, along with galactomannan and/or inulin fibres, may exert more potent anti‐obesity effects on account of synergism in terms of SCFA production and microbiota ‘re‐configuration’ (Hofmann et al., 2014). Administration of synbiotics during very early period in life, that is, in the postnatal period, has been shown to cause alterations in the gut microbiota of mice, which prevented the development of diet‐induced obesity later in life in these mice (Wegh et al., 2019). A recent meta‐analysis of 10 RCTs concluded that diets supplemented with either prebiotics or synbiotics may provide beneficial effects in terms of lipid metabolism and glucose homeostasis among patients with T2DM (Mahboobi et al., 2018).

### Exercise

Recent studies have shown that exercise can increase the number of beneficial microbial species, enrich microbiota diversity and promote the development of symbiotic bacteria. For instance, physical exercise was found to modulate the gut microbiome composition in a study; the abundance of health‐promoting bacterial species, including *F. prausnitzii*, *Roseburia hominis* and *A. muciniphila*, was found to be increased in physically active women compared with that in sedentary women (Bressa et al., 2017). Voluntary running was reported that caused changes in the microbiome composition, increase in the n‐butyric acid concentration and increase in caecum diameter (Matsumoto et al., 2008). In addition, Evans et al. (2014) reported that exercise prevents obesity and induces changes in the percentage of major phyla in mice with high‐fat diet‐induced obesity; the total distance covered was found to be inversely correlated with the Bacteroidetes:Firmicutes ratio. Thus, exercise plays an important role in the prevention of diet‐induced obesity, producing a microbial composition in obese mice similar to that of lean mice. The researchers reported that exercise provides a unique microbiome independent of diet. The abundance of *F. prausnitzii*, which protects the digestive tract by producing butyrate, was found to be increased in exercising mice (Campbell et al., 2016). Mika *et al*. (2015) observed that exercise during infancy resulted in modification of various phyla in the microbiome, with increased abundance of Bacteroides and decreased abundance of Firmicutes. Juvenile exercise modified more genera and led to an increase in lean body mass compared with adult exercise. These data suggest that early life exercise can influence the intestinal microbiome composition, stimulating the development of bacteria that promote host's metabolic fitness. Forced and voluntary exercise altered the microbiome in the caecum and faeces of mice, leading to variations in microbial taxonomy. Petriz et al. (2014) studied the effects of controlled exercise training on the intestinal microbiota of obese and hypertensive rats. They found that the intestinal microbiota composition of nonobese and hypertensive rats differed from that of the obese rats, which suggests that exercise regulates intestinal microbiota and may be recommended for the treatment of obesity and/or hypertension.

A study on humans showed that exercise enriched the intestinal floral diversity and was positively correlated with protein intake and creatine kinase levels. In addition, individuals with low BMI and athletes exhibited higher levels of *A. muciniphila* in their microbiota than those with high BMI (Clarke et al., 2014). These bacteria are slime‐degrading bacteria located in the mucus layer and are negatively correlated with BMI, obesity and metabolic disorders, which may be due to their role in improving the barrier function. These findings support the idea that intestinal microbiota exerts a health‐promoting effect on hosts in terms of energy balance and body composition during exercise.

## CONCLUSION

The species diversity and abundance of intestinal micro‐organisms affect energy absorption and regulate lipid metabolism in animals. Colonization of certain micro‐organisms such as *Faecalibacterium*, Lachnospiraceae, Ruminococcaceae and *Anaerofilum*, belonging to the Firmicutes, in the intestinal tract can lead to obesity, whereas the colonization of other microbes, such as Bacteroidetes, can make animals lean. In addition, the metabolites of intestinal micro‐organisms participate in lipid metabolism regulation in animals. For example, SCFAs regulate gene expression through corresponding receptors and maintains energy balance through the intestinal brain axis. BSH‐induced increase in hepatic bile acid synthesis may be mediated by Farnesoid X receptor, which plays an important role in regulating bile acid and lipid metabolism. FMO3 is a powerful regulator of cholesterol metabolism and RCT. LPS can affect lipid metabolism in mammals by converting conjugated bile acids into free bile acids. At the same time, different external factors and internal factors (exercise and diet) can change the intestinal microbiota and further affect the regulation mechanism of lipid metabolism.

## FUTURE DIRECTIONS

Extensive research on intestinal microbiota has clearly shown that intestinal micro‐organisms regulate lipid metabolism in the host through various complex mechanisms, such as by secreting metabolites. However, a knowledge gap remains in the mechanism and causality. Although much attention has been paid to SCFAs, the mechanism through which SCFAs regulate glucose metabolism and fat deposition after reaching the liver remains poorly understood. Most of the available data linking the microbiome to disease processes have been generated in animal models, which do not fully reflect the association of microbiome with human disease. Thus, animals feeding research needs to confirm their relevance before it can be translated into practical nutritional advice. Research on the function of intestinal microbiome in improving health and preventing diseases is still in the early stages. In‐depth research and rapid progress in studies on the intestinal microbiome highlight the potential and necessary perspectives to target the intestinal microbiome for the treatment and improvement of lipid metabolism‐related diseases. Understanding the metabolic interactions between the gut microbiota and the host is essential to develop personalized therapeutic strategies, including novel prebiotics, probiotics and synbiotics, to prevent or treat obesity, metabolic disorders and the associated cardiovascular diseases.

## CONFLICT OF INTEREST

No conflict of interest was declared.
